# Knowledge, Perception, and Performance of Hand Hygiene and Their Correlation among Nursing Students in Republic of Korea

**DOI:** 10.3390/healthcare9070913

**Published:** 2021-07-19

**Authors:** Hyang Soon Oh

**Affiliations:** Department of Nursing, Sunchon National University, Suncheon 57922, Korea; ohs2016@sunchon.ac.kr

**Keywords:** hand-hygiene, knowledge, perception, student, nursing

## Abstract

Recently, various outbreaks of newly emerging or reemerging diseases are expected more frequently and regularly. The importance of hand hygiene (HH) competency of nursing students (NS) is further required as a crucial learning objective of nursing education in universities. Purpose: This study aimed to investigate knowledge, perception, and performance of HH among NS and analyze their correlation. Methods: A cross-sectional questionnaire (modified from a World Health Organization questionnaire) was conducted from 23 November to 22 December 2019; 233 responses were used for the final analysis. Results: The average scores (mean ± standard deviation (range)) for knowledge, perception, and performance of HH were 17.82 ± 2.15 (0–25), 77.24 ± 10.78 (15–96), and 67.42 ± 23.10 (0–100), respectively. No significant variables were discovered to the knowledge of HH. Grade, university-affiliated hospitals, and the most recent healthcare institute of clinical practice nursing course significantly affected perceptions of HH (*p* < 0.039, *p* = 044, *p* < 0.001). Knowledge of HH was positively correlated with performance of HH (*p* = 0.002). The perception and the performance of HH of NS were positively correlated with HH performance of healthcare workers (HCWs); *p* < 0.001, *p* = 0.002. Conclusion: HH education for NS is crucial for improving the performance and the knowledge of HH. Good HH performance of healthcare workers (HCWs) can contribute to increased perception and performance of HH among NS. The cooperation of nursing education in a university and clinical practice with competent HCWs in healthcare institutions may create an effective education program for good HH performance of NS, who will be nurses during unpredictable pandemics.

## 1. Introduction 

Healthcare-associated infections (HAI) seriously impact morbidity and mortality, extend hospital stays, and burden hospital costs [1]. Therefore, infection control and prevention (ICP) are significant nursing care tasks. Recently, epidemics of newly emerging infectious diseases, such as severe acute respiratory distress syndrome, avian influenza, and Middle East respiratory distress syndrome (MERS), increased [2,3,4]. These newly emerging infectious diseases further emphasized the importance of ICP in nursing care [2]. 

Hands of healthcare workers (HCWs) are the primary transmission route of infections; hand hygiene (HH) is the simplest, most crucial method of ICP to prevent the spread of infections [4]. However, the performance of HH among HCWs was only 40–60% despite the significant role of HH for preventing infection transmission [4,5]. Given that nurses are the most frequent contacts of patients, their HH performance is the most important among HCWs [6,7].

As the value of HH in clinical nursing increased, the competency of nursing students (NS) in HH is required during nursing education in universities to ensure the future competency of nurses [8]. Therefore, studies regarding the current status of HH knowledge, perception, and performance among NS were first required [9]. It is therefore necessary to discover how HH knowledge, perception, and performance among NS are related to developing educational strategies for NS to improve HH performance competency [10,11,12]. In prior literature reviews, studies regarding the status of knowledge and performance of HH among NS were conducted actively [11,12]; however, studies regarding predictors of HH performance among NS were reported scarcely [10]. 

Additionally, in the Republic of Korea (hereafter Korea), the competency of HH as a means of ICP preparedness for infectious disease outbreaks is strongly required since the 2015 MERS outbreaks in hospitals [13]. Moreover, the need for excellent NS with high competence of HH is continuously increasing after the MERS outbreak in Korea. Furthermore, in the future, various outbreaks of newly emerging or reemerging diseases will be expected more frequently and regularly than before [2]; as such, the importance of HH may rapidly increase [1,3,4]. Accordingly, to prepare for unpredictable infectious disease epidemics, the importance of HH competency among NS is further required as a crucial learning objective of nursing education in universities. However, studies regarding HH were conducted less among NS than nurses. Further, a few studies using HH tools were published by the World Health Organization (WHO) [14,15]. Therefore, there were some limitations in comparing HH results among NS in Korea to those in other countries.

This study investigated the current status of HH knowledge, perception, and performance among NS in Korea after MERS outbreaks. Additionally, it analyzed significant variables and correlations among them for developing teaching strategies of HH in nursing education.

## 2. Methods

### 2.1. Participants 

Convenience sampling was conducted among four national universities in three rural areas in Korea. Inclusion criteria were being a junior and senior NS who took clinical practice nursing courses. Exclusion criteria were being a freshman or sophomore who had not yet taken clinical practice nursing courses. The minimum sample size, calculated using G*power version 3.1.9.4 (Franz Faul, Universitat Kiel, Kiel, Germany), was 207 (effect size = 0.25; power = 0.9, σ error probability = 0.05; groups = 3; F-test family for ANOVA statistics). The final sample size was 250, considering a possible non-response rate of over 20%. Participants voluntarily agreed and consented to the purpose of this study and participated voluntarily; they were informed that they could withdraw consent at any time during the study. 

### 2.2. Questionnaires 

Questionnaires had three parts—A, B, and C—to assess the participants’ knowledge, perceptions of HH, and general characteristics, respectively. Part A had 25 questions (multiple-choice, true or false, and yes or no), with each question scored as either 1 or 0 for right and wrong answers, respectively; the total score for this part was from 0–25 (Supplementary Table S1). Part B had 18 questions with 3 and 12 questions rated on 4 and 7-point Likert scales, respectively; the total range of scores for perception was 15–95 (Supplementary Table S2). The remaining three questions from Part B (B1, B5, B11) were self-reports of the HAI rates, the performance of HH among HCWs, and the performance of HH among NS. The higher the scores for knowledge, perception, or performance were, the higher the participants’ knowledge, perceptions, or performance was. Finally, Part C had 13 questions on age, sex, year level, HH resources (sinks for HH, alcohol-based hand sanitizer) in the laboratory of a nursing university, the most recent healthcare institute of clinical practice nursing course, major nursing course for HH education, the experience of HH education within the last year, regular use of alcohol-based hand sanitizer, and nursing courses important to increasing knowledge and perception of HH. 

This questionnaire (Parts A and B) was revised from questionnaires that modified WHO tools [14,15]. Further, they were used in previous studies [16,17]. The pilot study was conducted with 20 NS; they were recruited from a university on 5 November 2019. The validity was confirmed by junior and senior NS of a nursing university, while a pilot study confirmed the reliability. The Cronbach’s α in the pilot study were 0.518 and 0.807 for knowledge (Part A) and perception (Part B), respectively; for the current study, the Cronbach’s α were 0.557 and 0.894 for knowledge (Part A) and perception (Part B), respectively. Finally, in a previous study, Cronbach’s α were 0.611 and 0.932 for knowledge (Part A) and perception (Part B), respectively [16].

### 2.3. Data Collection 

A cross-sectional survey using questionnaires was conducted. Documented informed consent to participate in this study was obtained from four national universities in three rural areas in Korea. Nursing faculties interested in this study voluntarily agreed to participate and for their data to be collected. Thereafter, consent to participate in this study was obtained from students in the above universities sequentially. Data were collected from 23 November to 22 December 2019 through anonymous, self-reported questionnaire surveys that took 10–15 min to answer. A total of 250 questionnaires were mailed to the participants with returned envelopes. Finally, 239 responses were returned (95.6%) after three reminders to mail the questionnaires. Only 233 were included in the final analysis, excluding six incomplete questionnaires. Nursing students were guaranteed voluntary and anonymous participation, and they were informed that they could withdraw from the study at any time. They received a predetermined token for participation in this study. 

### 2.4. Ethics

The Institutional Review Board of Sunchon National University (104173-201911-HR-036-02) approved this study. 

### 2.5. Analysis 

Data were analyzed using SPSS (version 24.0: IBM Corp., Armonk, NY, USA). Cronbach’s α was calculated. Descriptive analysis was used for general characteristics, knowledge, perception, and HH performance, while univariate analysis was used to determine differences among these relative to general characteristics. Pearson’s correlation analysis was also conducted between knowledge, perception, and performance. Knowledge, perception, and performance of HH were described by the mean ± standard deviation (SD) and maximum and minimum values. Knowledge was categorized into three rates according to the correct answer rate: high (≥90%), medium (70.0–89.0%), and low (<70.0%) [17]. Knowledge was considered acceptable if it was scored as medium level and over. The perception was considered acceptable if scores were ≥3 or ≥5 on 4- and 7-point Likert scales, respectively. Data were not normally distributed (Shapiro–Wilk, *p* < 0.001), thus non-parametric analyses were conducted through Mann–Whitney U or Kruskal–Wallis tests. Finally, *p*-values (*p*) < 0.05 were considered significant.

## 3. Results

### 3.1. General Characteristics 

There were 233 participants (*n* = 233) from four national universities in three rural areas, with there being 50 (21.5%), 45 (19.3%), 63 (27.0%), and 75 (32.2%) participants, respectively. Most participants were females (82.0%), seniors (71.7%), and university-affiliated hospitals (40.8%). HH resources—sinks for handwashing and alcohol-based hand sanitizer—were installed in 99.6% of the nursing practice rooms of nursing schools. The most recent healthcare institute individuals took clinical practice nursing courses in were advanced general hospitals (46.8%). HH education was performed through various classes, such as theoretical, laboratory practical, and clinical practice nursing courses. Most participants (88.8%) completed HH education through nursing education courses, while 51.1% completed HH education courses via mass media. Most participants (74.2%) answered they regularly used alcohol-based hand sanitizer. Participants answered that the most important nursing courses for HH knowledge and perception improvements were laboratory (37.8%) and clinical (44.2%) practice nursing courses, respectively. Details of general characteristics are described in Table 1.

### 3.2. Knowledge, Perception, and Performance of HH

**Figure 1:**
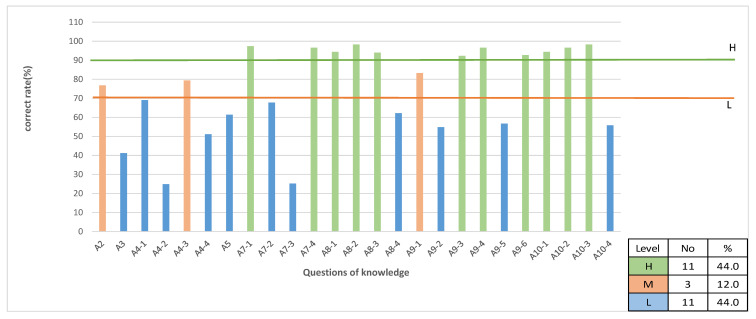
Correct answer rate of each question of knowledge regarding hand hygiene among nursing students. Level H, high (90% and over); M, medium (70–89%); L, low (69% and below).

The average score for knowledge was 17.82 ± 2.15 (mean ± SD) (range of 0~25). The average correct answer rate of knowledge questions (%) was 74.46 ± 23.32 (mean ± SD). A total of 56% of knowledge questions showed medium or high correct answer rates, and 44.0% of knowledge questions were classified as “low” levels (Figure 1). Knowledge items with low correct answer rates—<50% correct answers—were the following: “What is the most frequent source of germs responsible for healthcare-associated infections (A3)?”, “Hand rubbing causes skin dryness more than handwashing (A4-2)”, “Handwashing and hand rubbing are recommended to be performed in sequence (A4-4)”, and “After exposure to immediate surroundings of a patient (A7-3)” (Figure 1).

**Figure 2:**
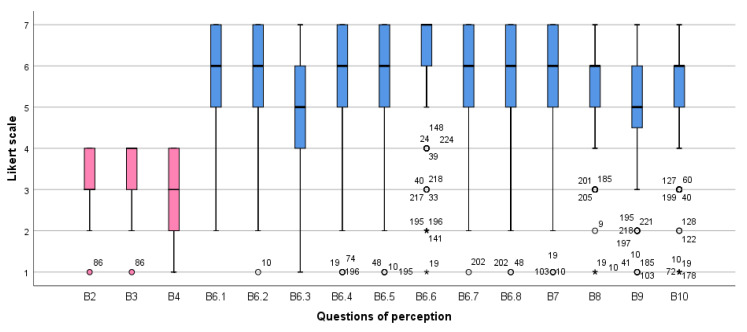
Box plot of perception of hand hygiene among nursing students (B2~B4, 4-point Likert scale (1–4); the others, 7 -point Likert scale (1–7)).

The average perception score was 77.24 ± 10.78 (mean ± SD) (range of 15~96). Perception showed a low score for item B4: “Among all patient safety issues, how important is hand hygiene at your institution of the most recent clinical practice course?” The lowest perception (*M* = 5.06 ± 1.58), despite being over the acceptable level (≥5), was for items B6.3: “Hand hygiene posters are displayed at the point of care as reminders”, B8: “What importance do your colleagues attach to the fact that you perform optimal hand hygiene?”, B9: “What importance do patients attach to the fact that you perform optimal hand hygiene?”, and B10: “How do you consider the effort required by you to perform good hand hygiene when caring for patients?” (Figure 2).

Questions of B1, B5, and B11 were excluded from the scores of perceptions because they were analyzed separately as HAI rate, HH performance among HCWs, and HH performance among NS, respectively. The performance of HH (%) among NS was 67.42 ± 23.10 (mean ± SD) on average. Participants’ scores for HAI (%) and HH performance (%) among HCWs were 45.79 ± 23.58 and 69.13 ± 21.90 (mean ± SD), respectively.

### 3.3. Differences and Correlation among Knowledge, Perception, and Performance of HH

Significant variables were not found on non-parametric analysis. However, HH’s perception among NS was significantly higher in senior students, those in a university with university-affiliated hospitals, and the type of most recent healthcare institute of clinical practice course. Moreover, the performance of HH among NS was significantly higher in males (Table 2). Meanwhile, on Pearson’s correlation analysis, knowledge and perception were not significantly correlated. Conversely, knowledge and performance were positively correlated. Perception and performance of HH among NS also showed a positive correlation with the performance of HH among HCWs (Table 3).

## 4. Discussion 

This study clarified the current status of HH’s knowledge, perception, and performance among NS after MERS in Korea, and further sought to find out significant factors and their correlations, which are useful for nursing education increasing knowledge, perception, and HH performance among NS regarding HH competencies.

Knowledge in this study was higher than in previous studies using WHO tools [14,15]: it was reported as 13.20 [18], 13.57 [12], 11.0 [19], and 15.86 [20]. This study’s average correct answer rate was similar to that (75%) of previous studies [12]. The medium level of knowledge of HH in this study was not optimal. As such, this suggests that HH knowledge in nursing education in universities must be strengthened. Moreover, questions that had low rates of correct answers, especially those with rates under 50%, should be prioritized in nursing education to improve HH knowledge. In this study, knowledge was found to be strongly correlated to the performance of HH. Therefore, the value of HH education and training in nursing universities for improving the performance of HH cannot be overemphasized [21].

Perception in this study was higher than in previous studies using WHO tools [14,15]: it was reported as 35.55 [19] and 5.62–5.82 [20]. However, participants in this study showed low perception for item B4 (“Among all patient safety issues, how important is HH at your institution of the most recent clinical practice course?”). This suggests that the importance of HH in healthcare instructions was not optimal. Therefore, continuous improvement efforts within healthcare institutions are required. Additionally, relatively low scores were also found for item B6.3 (“Hand hygiene posters are displayed at the point of care as reminders”), similar to previous studies [22]. Therefore, to improve the perception of HH in general, it would be beneficial to adopt methods recognized by participants as effective, such as those in items B6.2 (“The health care facility makes alcohol-based hand rub always available at each point of care”), B6.6 (“Health care workers regularly receive feedback on their hand hygiene performance”), and B6.8 (“Patients are invited to remind healthcare workers to perform hand hygiene”).

This study showed that clinical practice nursing courses in an advanced general hospital could improve the perception of HH among NS. Furthermore, male students showed higher perception and performance than female students in this study, similar to previous findings [18]. This finding may appear to influence the preference for male nurses positively. However, there are limitations to forming conclusions with this study alone; thus, further research is required.

This study discovered valuable information through correlation analysis; the performance of HH among NS significantly correlated with knowledge of HH among NS. Additionally, the performance of HH and the perception of HH among NS were significantly correlated to HH’s performance among HCWs of the most recent healthcare institute of clinical practice nursing course. This result corresponded to previous results [23,24,25]. Thus, the performance of HH among HCWs can contribute to the performance and the perception of HH among NS. Therefore, good role models of HH performance among HCWs can significantly affect the perception and the performance of HH among NS, suggesting that cooperation of nursing universities and health care institutions regarding HH education for NS is of value. This cooperation of both theoretical educations of HH in nursing universities and practical education of HH in healthcare institutions will be very effective educational programs for the HH competency of nursing students. 

Furthermore, training NS with competent HH is vital for this era of antimicrobial resistance to prevent HAI with multidrug resistance for bacterial evolution [26] and to address the use of antimicrobial disinfectants against multidrug-resistant bacteria and viruses and clinical applications as detergents in surgery. In the clinical setting, it is recommended to prevent the spread of HAIs with antimicrobial resistance such as Staphylococcus and Pseudomonas aeruginosa [27] and effective decontamination of coronavirus [28]. Therefore, HH using these kinds of antiseptics and topical application is effective for HH, especially against coronavirus [29].

At the time of writing this paper, there are severe restrictions in the clinical practice curriculum of NS in healthcare institutions due to social distancing caused by the coronavirus disease-2019 (COVID-19) pandemic. Thus, this restriction in clinical practice nursing courses in healthcare institutions can impact the perception or the performance of HH among NS. Therefore, further research is required. Moreover, further research regarding alternatives to clinical practice nursing courses in healthcare institutions, for example, developing a simulation curriculum during the social distance of the COVID-19 pandemic, is required.

One limitation of this study is that participants only came from four universities in some rural regions in Korea; as such, generalization of the results to all NS in Korea is not possible. Additionally, HAI rates and performance of HH were self-reported. Thus, these figures may be overestimated. However, this study found improvement of knowledge, perception, and performance of HH among NS after the MERS outbreak, weaknesses in the knowledge that can be improved and reflected in nursing education in universities, and a significant positive correlation between HH’s performance among NS and HCWs. 

## 5. Conclusions

Knowledge of HH among NS can impact performance; low HH knowledge levels may affect the low performance of HH. Therefore, improvement of knowledge is first required to improve performance in terms of HH competency. Strengthening education on HH during nursing courses in universities is needed for the improvement of HH knowledge. HH performance among HCWs of the healthcare institutions can be role models of HH performance among NS. Therefore, close cooperation between nursing colleges and clinical practice institutions should be further strengthened. HH education programs for NS with competent HCWs during clinical practice courses at healthcare institutions are effective for improving HH performance of NS. This cooperation may support preparations for HH competence among NS, who are nurses during unpredictable pandemics. 

## Figures and Tables

**Table 1:** General characteristics of participants (*n* = 233).

Variables	*n* (%) or Mean ± SD
**Age (year)**	22.43 ± 1.53
**Sex**	
Male	42 (18.0)
Female	191 (82.0)
**Year level**	
Junior	63 (27.0)
Senior	167 (71.7)
Missing	3(0.3)
**University-affiliated hospital**	
Yes	95 (40.8)
No	138 (59.2)
**Hand hygiene resources in a laboratory in a nursing university**	
Sink for hand hygiene	232 (99.6)
Alcohol-based hand sanitizer	232 (99.6)
Missing	1(0.4)
**The most recent healthcare institute of clinical practice nursing course**	
Advanced general hospital	109 (46.8)
General hospital	32 (13.7)
Hospital	24 (10.8)
Community-based healthcare center	33 (14.2)
Missing	35(15.0)
**Major nursing course of hand hygiene education (multiple-choice)**	
Theoretical nursing course	108 (46.4)
Laboratory practice nursing course	125 (53.6)
Clinical practice nursing course	143 (61.4)
Received hand hygiene education within the last year via a nursing course	207 (88.8)
Received hand hygiene education within the last year via mass media	119 (51.1)
Regular use of alcohol-based hand sanitizer	173 (74.2)
**Essential nursing courses to increase knowledge of hand hygiene (multiple-choice)**	
Theoretical nursing course	35 (15.0)
Laboratory practice nursing course	88 (37.8)
Clinical practice nursing course	82 (35.2)
Mass media	10 (4.3)
**Essential nursing courses to increase perception of hand hygiene (multiple-choice)**	
Theoretical nursing course	37 (15.9)
Laboratory practice nursing course	70 (30.0)
Clinical practice nursing course	103 (44.2)
Mass media	8 (3.4)

**Table 2:** Non-parametric analysis (*n* = 233).

Variables			Mean ± SD	N	*p*-Value *
**Perception**	**Sex**	Male	80.17 ± 10.31	42	0.051
		Female	76.61 ± 10.98	191	
	**Grade**	Junior	74.67 ± 12.30	63	0.039
		Senior	78.49 ± 9.78	167	
	**University-affiliated hospital**	Yes	79.71 ± 10.32	95	0.044
		No	75.57 ± 11.04	138	
	**The most recent healthcare institute of clinical practice nursing course**	Advanced general hospital	79.95 ± 10.85	109	<0.001 *
		General hospital	76.06 ± 9.85	32	
		Hospital	69.96 ± 12.32	24	
		Community-based public healthcare center	76.12 ± 7.82	33	
**Performance**					
	**Sex**	Male	75.95 ± 19.29	42	0.004
		Female	65.74 ± 23.44	183	

Non-parametric univariate analysis (Mann–Whitney; * Kruskal–Wallis).

**Table 3:** Pearson’s correlation analysis.

Variables	Knowledge	Perception	Performance	Performance of Health Care Workers
Knowledge	1			
Perception	0.036	1		
Performance	0.210 **	0.096	1	
Performance of health care workers	0.123	0.469 **	0.220 **	1

** *p* < 0.001; two-tailed by Pearson’s correlation analysis.

## Data Availability

The data that support the findings of this study are available from the corresponding author upon reasonable requests.

## Supplementary Materials

The following are available online at https://www.mdpi.com/article/10.3390/healthcare9070913/s1, Table S1. Questions of Knowledge of Hand hygiene. Table S2. Questions of Perception of Hand hygiene.
