# Supplementary material for: Knowledge, Perception, and Performance of Hand Hygiene and Their Correlation among Nursing Students in Republic of Korea
Source: Healthcare (Basel). 2021 Jul 19;9(7):913. doi: 10.3390/healthcare9070913 (PMC8304706; doi:10.3390/healthcare9070913)
Supplement: Supplementary file 1 [file healthcare-09-00913-s001.zip › healthcare-1266828-supplementary.pdf]

Supplementary Tables

Supplementary Table 1. Questions of Knowledge of Hand hygiene

		Questions	Correct answer
**A2**		Which of the following is the main route of transmission of potentially harmful germs between patients?	Health care workers' hands when not clean
**A3**		What is the most frequent source of germs responsible for health care-associated infections?	Germs already present on or within the patient
**A4**		Which of the following statements about alcohol-based hand rub and handwashing with soap and water is true?	
	**A4-1**	Hand rubbing is more rapid for hand cleansing than handwashing.	True
	**A4-2**	Hand rubbing causes skin dryness more than handwashing.	False
	**A4-3**	Hand rubbing is more effective against germs than handwashing.	False
	**A4-4**	Handwashing and hand rubbing are recommended to be performed in sequence.	False
**A5**		What is the minimal time needed for alcohol-based hand rub to kill most germs on your hands?	20 seconds
**A7**		Which of the following hand hygiene actions prevents transmission of germs to the patient?	
	**A7-1**	Before touching a patient	Yes
	**A7-2**	Immediately after risk of body fluid exposure	Yes
	**A7-3**	After exposure to immediate surroundings of a patient	No
	**A7-4**	Immediately before a clean/aseptic procedure	Yes
**A8**		Which of the following hand hygiene actions prevents transmission of germs to the health care worker?	
	**A8-1**	After touching a patient	Yes
	**A8-2**	Immediately after a risk of body fluid exposure	Yes
	**A8-3**	Immediately before a clean/aseptic procedure	No
	**A8-4**	After exposure to the immediate surroundings of a patient	Yes
**A9**		Which type of hand hygiene method is required in the following situations?	
	**A9-1**	Before palpation of the abdomen	Rubbing
	**A9-2**	Before giving an injection	Rubbing
	**A9-3**	After emptying a bed pan	Washing
	**A9-4**	After removing examination gloves	Rubbing/Washing
	**A9-5**	After making a patient's bed	Rubbing
	**A9-6**	After visible exposure to blood	Washing
**A10**		Which of the following should be avoided, as it is associated with an increased likelihood of the colonization of hands with harmful germs?	
	**A10-1**	Wearing jewelry	Yes
	**A10-2**	Damaged skin	Yes
	**A10-3**	Artificial fingernails	Yes
	**A10-4**	Regular use of a hand cream	No

Supplementary Table 2. Questions of Perception of Hand hygiene

Perception			Questions
**Health care-associated infection (Range 0–100%)**	B1*		In your opinion, what is the average percentage of hospitalized patients who will develop a health care-associated infection (between 0 and 100%)?
	B2†		In general, what is the impact of a health care-associated infection on a patient's clinical outcome?
	B3†		What is the effectiveness of hand hygiene in preventing health care-associated infection?
	B4†		Among all patient safety issues, how important is hand hygiene at your institution?
**Hand hygiene performance of healthcare workers (Range 0–100%)**	B5*		On average, in what percentage of situations requiring hand hygiene do health care workers in your hospital actually perform hand hygiene, either by hand rubbing or handwashing (between 0 and 100%)?
	B6‡		In your opinion, how effective would the following actions be to improve hand hygiene permanently in your institution?
		B6.1	Leaders and senior managers at your institution supporting and openly promoting hand hygiene
		B6.2	The health care facility makes alcohol-based hand rub always available at each point of care.
		B6.3	Hand hygiene posters are displayed at point of care as reminders.
		B6.4	Each health care worker receives education on hand hygiene.
		B6.5	Clear and simple instructions for hand hygiene are made visible for every health care worker.
		B6.6	Health care workers regularly receive feedback on their hand hygiene performance
		B6.7	Always performing hand hygiene as recommended (as a good example to colleagues).
		B6.8	Patients are invited to remind health-care workers to perform hand hygiene.
	B7‡		What importance does the head of your department attach to the fact that you perform optimal hand hygiene?
	B8‡		What importance do your colleagues attach to the fact that you perform optimal hand hygiene?
	B9‡		What importance do patients attach to the fact that you perform optimal hand hygiene?
	B10‡		How do you consider the effort required by you to perform good hand hygiene when caring for patients?
**Hand hygiene performance of self (Range 0- 100%)**	B11*		On average, in what percentage of situations requiring hand hygiene do you actually perform hand hygiene, either by hand rubbing or handwashing (between 0 and 100%)?

* Questions of B1, B5, and B11 were excluded from the scores of perceptions because they were analyzed separately as healthcare associated infection rate, hand hygiene performance of healthcare workers, and hand hygiene performance of the nursing student, respectively.

† 4-point Likert scale (1–4); ‡ 7 -point Likert scale (1–7)
